# Editorial: Women in insect science, volume II

**DOI:** 10.3389/finsc.2026.1847113

**Published:** 2026-04-23

**Authors:** Immacolata Iovinella, Emma J. Hudgins, Mariana Bulgarella, Sudeshna Mazumdar-Leighton

**Affiliations:** 1 Council for Agricultural Research and Economics – Research Centre for Plant Protection and Certification (CREA-DC), Research Centre for Plant Protection and Certification, Firenze, Italy; 2 The University of Melbourne School of Agriculture Food and Ecosystem Sciences, Parkville, Australia; 3 Victoria University of Wellington School of Biological Sciences, Wellington, New Zealand; 4 Department of Botany University of Delhi, Delhi, India

**Keywords:** applied and basic entomology, gender diversity, diversity and inclusion in STEM, scientific role models, women in entomology

## 1 Introduction

The *Women in Insect Science* Research Topic series aims to highlight and celebrate the scientific contributions of women in entomology—an area of research that remains crucial for global and local biodiversity, agriculture, ecosystem functioning, and public health. Despite the centrality of entomological research to many societal challenges, women remain underrepresented in senior positions, occupying less than 25-27% of academic and federal entomology jobs, according to new research published in the *Annals of the Entomological Society of America*. The situation is particularly stark in the global south where women remain marginalized in leadership roles in STEM-based fields. This creates and perpetuates a lack of relatable role models for young scholars, exacerbated by parochial perceptions, societal norms and lack of precedence for women leading research and enterprise in insect science. This Research Topic was developed precisely to provide visibility, recognition, and support to women scientists, while also offering inspiration for future generations of female entomologists.

This second volume brings together four contributions authored or led by women researchers, spanning diverse areas of insect science—from physiology and development to behavior, neurobiology, and applied entomology. Although limited in number, the articles included in this Research Topic illustrate the breadth and depth of contemporary research conducted by women in the field, and they exemplify the value of promoting diverse perspectives within the scientific community. Here, we provide an overview of these contributions and place them within the broader context of current entomological research.

## 2 Overview of contributions

### 2.1 Insect physiology and development

Our Research Topic includes two articles investigating the role of insect diet in key demographic rates, with applications to cost-effective, scalable insect rearing.

The first is Original Research investigating the world’s most economically damaging invasive species by recent estimates (1), *Aedes aegypti - a* vector of diseases like Yellow Fever. The authors assess the role of larval diet in rearing mosquitoes infected with a common bacterial control agent, Wolbachia (Gunasekaran et al.). Wolbachia-based control requires successful mass rearing and release of Wolbachia-infected larvae. Fish feed was the most effective larval diet, primarily concluded based on mosquitoes maintaining high fecundity on this diet after Wolbachia infection. We also include an Original Research article that investigates the storage requirements for artificial insect diets once ‘completed’ i.e., ready to use (Ho et al.). Insect weight and survival of western corn rootworm (*Diabrotica virgifera virgifera* LeConte) larvae were maintained only with cold storage below 4 °C after 24hr of diet storage, though all completed diets declined in quality after seven days of storage.

### 2.2 Neurobiology and behavioral ecology

The next article in our Research Topic is a Mini Review of the role of dopamine in foraging circuitry (Ye et al.). While their focus is social insects, the review also draws on learning from across taxa – concentrating on conserved neuroanatomy. Social insects produce emergent foraging patterns from smaller-scale individual insect decisions analogously to vertebrate behaviours such as bird collective escape (2). The authors describe preliminary work connecting dopamine to environmental conditions and suggest a key research gap in how this circuitry may be impacted by climate change.

### 2.3 Forensic entomology and applied sciences

The final article in our Research Topic is a Review synthesizing the effects of narcotic drugs on the reliability of insects used in forensic analyses to estimate approximate time of death (Jain et al.), known as post-mortem interval (3). While the final stages of insect lifecycles are generally unaltered by these drugs, pupation is commonly delayed due to drug exposure. Given the variability in larval timing impacts of drugs across insect species, the authors recommend a more comprehensive assessment of narcotics on all important forensic entomology species.

## 3 Role models in entomology

In addition to showcasing contemporary research led by women, this Research Topic can be viewed within a broader historical context shaped by pioneering women whose work laid important foundations for modern entomology. Historically, entomology has been a heavily male-dominated field. Here we pay tribute and celebrate two pioneering and visionary women who achieved excellence in entomology.

Professor Mary Jane West-Eberhard is a renowned entomologist and evolutionary biologist whose work on tropical social wasps has had a profound impact on evolutionary theory. Trained at the University of Michigan, she later conducted much of her research at the Smithsonian Tropical Research Institute. Her influential book *Developmental Plasticity and Evolution* reshaped thinking on the role of phenotypic plasticity in evolutionary processes. Beyond her scientific achievements, she has been an advocate for women (and mothers) in entomology, setting an important example of scientific excellence combined with mentorship and inclusivity.

Professor Aola Richards (1927–2021) was the first woman in New Zealand to earn a PhD in Biological Sciences in 1958. Her research on cave-dwelling insects and ladybird beetles bridged entomology and speleology and led to the description of more than 20 new species and more than 80 published papers. Richards also left generous bequests to advance insect research at three research institutions established in New Zealand, Australia, and the United Kingdom.

The admirable careers of Mary Jane West-Eberhard and Aola Richards exemplify scientific rigor, curiosity, and resilience, qualities that continue to characterize the work of women in entomology today. We hope that their stories will inspire the next generation of women in entomology.

## 4 Future directions

The contributions gathered here open several avenues for future work. As global challenges related to climate change, vector-borne diseases, food security, and ecosystem resilience intensify, entomology will remain a critical scientific discipline. Strengthening gender diversity within this field is not only a matter of equity but also a driver of scientific innovation. Research Topics such as this one serve as platforms to amplify valuable contributions, foster collaborations, and inspire new generations of women researchers.

We hope that future editions of this Research Topic will attract an even wider range of topics and approaches, continuing to reflect the creativity, rigor, and commitment of women in insect science.

## 5 Conclusion

We thank all authors, reviewers, and editors who contributed to this Research Topic. Their work not only advances entomological research but also helps build a more inclusive scientific community. We hope this second volume of *Women in Insect Science* hosted by Frontiers in Insect Science will serve as a source of inspiration and a catalyst for continued progress in promoting gender diversity and scientific excellence in entomology.
